# Ultrasound-guided fan biopsy of the breast: clinical utility and outcomes from a single-center experience

**DOI:** 10.1093/bjro/tzag013

**Published:** 2026-08-22

**Authors:** Ramona Menezes, Ruchira Sinnatamby, Helen Taylor, Nuala A Healy

**Affiliations:** Nottingham Breast Institute, Nottingham University Hospitals NHS Trust, Nottingham, NG5 1PB, United Kingdom; Department of Radiology, University of Cambridge, Cambridge CB2 0QQ, United Kingdom; Cambridge Breast Unit, Cambridge University Hospitals NHS Foundation Trust, Addenbrookes’ Hospital, Cambridge, CB2 0QQ, United Kingdom; Cambridge Breast Unit, Cambridge University Hospitals NHS Foundation Trust, Addenbrookes’ Hospital, Cambridge, CB2 0QQ, United Kingdom; Cambridge Breast Unit, Cambridge University Hospitals NHS Foundation Trust, Addenbrookes’ Hospital, Cambridge, CB2 0QQ, United Kingdom; Department of Radiology, Beaumont Hospital, Dublin, D09 V2N0, Ireland; Department of Radiology, Royal College of Surgeons, Dublin, D02 YN77, Ireland

**Keywords:** breast, ultrasound, breast biopsy

## Abstract

**Objective:**

US-guided non-focused “fan” biopsy of the breast allows for wider sampling directed to a more regional, less focal abnormality seen on US, MRI, mammogram or to an area of clinical concern. The aim of this study was to evaluate our experience of using this technique which has received little formal literature attention.

**Methods:**

A retrospective service evaluation was conducted of all US-guided fan biopsies of the breast at a single center between January 2015 and December 2021 with review of indications for use, histological outcomes, subsequent patient management and whether it optimized the patient pathway by enabling timely histological diagnosis, avoiding delays associated with scheduling x-ray or MRI-guided biopsies, and facilitating treatment decisions.

**Results:**

90 ultrasound-guided fan biopsies were performed in 89 patients, median age 52.6 years (range 29-90 years) over 7 years. In 49% of cases, the indication was non-mass enhancement seen on MRI. Biopsies yielded malignancy in 31% of cases and this technique was found to be helpful in obtaining histology in 93% (84/90) cases with false negative/inconclusive results in 6.6%.(6/90 cases).

**Conclusion:**

Our results suggest the relatively simple procedure of US-guided breast fan biopsy is a cost effective, versatile technique with potential to add value in the patient’s pathway.

**Advances in knowledge:**

US-guided fan biopsy is a pragmatic method to biopsy breast lesions that are not amenable to conventional targeted biopsy, or require more invasive and expensive techniques, and thus can help avoid delays in the patient diagnostic pathway and facilitate treatment decisions.

## Introduction

Imaging plays a key role in the diagnosis of breast lesions, with image-guided minimally invasive techniques now the gold-standard for obtaining tissue diagnosis. While x-ray guided, US-guided, and MR-guided biopsies are all established methods of targeted percutaneous breast biopsy, US guidance is preferred due to advantages of patient comfort, lack of ionizing radiation, readily available equipment, accessibility of all areas, real-time needle visualization, and low cost.^1–3^

Due to these advantages, attempts are generally made to identify focal US correlates for most breast lesions warranting biopsy.^2^ However, in routine practice we are often faced with situations when a lesion detected clinically or by other modalities such as mammogram or MRI is not sonographically evident as a focal lesion at second-look US, but rather as a diffuse area of altered echogenicity. Challenges arise when such lesions cannot be readily biopsied using alternative methods of guidance. There are instances when x-ray and MRI-guided biopsies cannot be safely performed, for example, due to the location of the lesion, proximity to the chest wall, patient intolerance, or thin breasts.^3^ Furthermore MRI-guided breast biopsy requires specific equipment, experienced, well-trained staff, and is time-consuming, expensive and only performed in a limited number of dedicated tertiary referral centers leading to potential delays in diagnosis.^4^

In such situations, where there is no discrete target, US-guided fan biopsy of the breast is a pragmatic approach with potential to obtain representative tissue as it offers it allows for a wider “fan shaped” sampling of the breast directed to a more regional abnormality in contrast to conventional biopsy which is targeted at a focal US abnormality. This technique involves covering an arc of the breast with multiple consecutive passes through the breast, with successive minor adjustments to the direction of needle. This allows sampling of a wider area through a single incision and can target diffuse abnormalities initially seen on MRI, mammography or areas of palpable clinical concern.

To the best of our knowledge, there is limited literature on the use and effectiveness of US-guided fan biopsy and no established guidelines. We aim to explain this technique and evaluate our experience by reviewing our local indications, histology, subsequent procedures and patient outcomes in order to determine whether this approach can provide value in the patient’s diagnostic pathway.

## Methods

This study was registered as a service evaluation with approval from the Clinical Audit team at Addenbrookes Hospital, Cambridge. As this was an observational study without intervention, the requirement for formal ethics approval and individual patient consent was waived.

### Study population

A search of US-guided breast procedures performed at Addenbrookes Hospital, Cambridge from January 2015 to December 2021 was undertaken using the hospital radiology information system to identify US-guided fan biopsies of the breast. Electronic patient records and patient archiving and communication systems for each of these cases were reviewed. Anonymized data was collected on patient demographics, mode of presentation (screening vs symptomatic), investigations prior to the fan biopsy and indication. Histology results, subsequent investigations/management after the fan biopsy and eventual patient outcomes were reviewed. No cases were excluded from this evaluation, so any potential bias would have arisen at the point of biopsy, which was performed based on clinical need.

All fan biopsy procedures were undertaken by 1 of 9 consultant breast radiologists, a consultant mammographer or a senior trainee undergoing fellowship training in breast imaging with direct consultant supervision. Regular team discussion and training ensured standardization of the technique described below.

**Figure 1:**
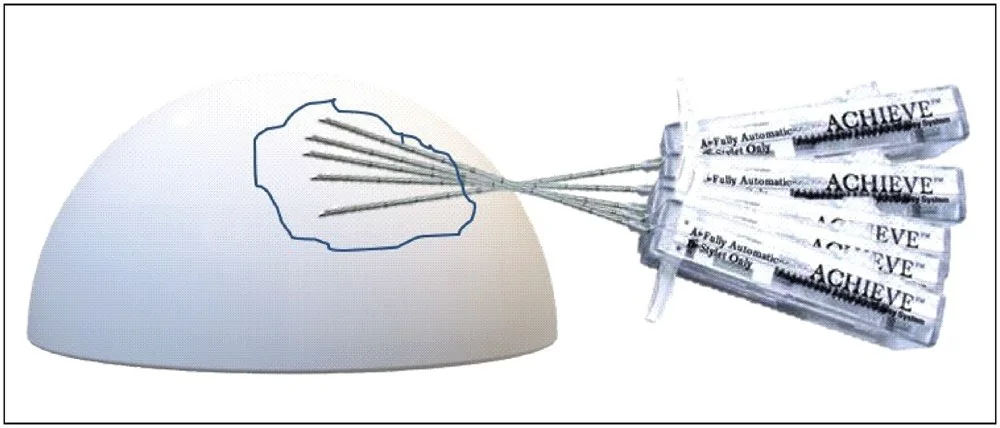
Illustration of the fan biopsy technique demonstrating a single skin incision and multiple core biopsies obtained in an arc through the outlined area of concern, via the single skin incision.

Technique of US-guided fan biopsy of the breast (Figure 1).

In each case, the indication for fan biopsy and all prior imaging investigations were reviewed to aid accurate biopsy planning, particularly important in cases targeting an abnormality visualized only on MRI. Informed verbal consent for the biopsy was obtained.

Once the area to be targeted by fan biopsy was identified, the optimal patient position (either supine or supine oblique) was chosen. Following aseptic technique, the skin was infiltrated with 3-6 mL of 1% lidocaine. The appropriate incision site was chosen with care, keeping in mind that this point will be the center from which consecutive core biopsies radiate out along an arc. The biopsy needle was introduced, directed at the center of the abnormality and first core obtained. The needle was then withdrawn, the sample retrieved, and needle reintroduced through the same incision, angled in slightly different direction so as to ensure wider sampling.

Multiple core biopsies were undertaken through the same incision site altering the angle of sampling by small increments so as to sample a wider fan shaped area. In our practice, between 3 and 6 cores of tissue were obtained according to patient tolerance. The number of passes should be sufficient to ensure accurate representation of the area of concern.

All biopsies were performed with a 14 or 16 G automated biopsy needle (Achieve, Carefusion, Vernon Hills, IL, United States). A Hydromark marker clip was deployed at the site of the biopsy in the center of the sampled area. Post biopsy care is the same as with any core biopsy, that is, pressure is applied for a few minutes followed by steri-strips and sterile dressing. Post procedure check mammogram was obtained to confirm accuracy of clip deployment and facilitate localization should the histology yield malignancy. In selected cases, a single non contrast T2 W MRI sequence was performed to confirm accuracy of clip placement where the indication was to target abnormality seen on MRI.

### Subsequent management

The results of all fan biopsies were discussed at a breast multidisciplinary team (MDT) meeting. Results with proven normal/benign result which were deemed concordant were discharged. B3 results^5^ underwent further sampling by vacuum assisted excision (VAE) or diagnostic excision as agreed by MDT discussion. Lesions deemed B4/B5 on histology underwent surgical management. If lesions were considered discordant at MDT, further imaging or biopsy was warranted.

Benign outcomes were defined by concordant benign histology or stability/resolution on follow-up imaging. The overall sensitivity and specificity were calculated on basis of whether the fan biopsy yielded histology results that prompted the appropriate management.^6^ False negatives were cases with benign fan biopsy results but subsequent malignant diagnosis. B3/B4 lesions later confirmed as malignant were considered true positives, while B3 lesions not upgraded at excision were classified as false positives. Two non-diagnostic cases (absence of microcalcification on specimen radiography) were also classified as false positives.

### Data collection and storage

Data were collected, anonymized, and stored in a password-protected Excel workbook. Analysis was performed using Excel and SPSS (version 26.0).

## Results

Ninety ultrasound-guided fan biopsies were performed in 89 patients between January 2015 and December 2021. The mean patient age was 52.6 years (range 29-90 years). 69 cases were symptomatic (77%) and 21 were screen-detected (23%) (Table 1). Eighty-eight patients were female, and one was male.

**Table 1:** Patient demographics.

Parameter	Number
**Average patient age**	52.6 years (range 29-90 years)
**Presentation type**	
**Symptomatic**	69 (76.6%)
**Screening**	21 (23.3%)

Indications for fan biopsy: Table 2 lists the indications for fan biopsies performed during the study period and subsequent yield of malignancy.

**Table 2:** Indications for US-guided fan biopsy breast and subsequent yield of malignancy.

Indications for performing fan biopsy	Number of cases	Malignancy rate on histology
**Targeting NME on MRI**	44 (48.88%)	11/44 (25%)
**Clinical concern with only vague or no US findings**	21 (23.33%)	7/21 (33.33%)
**Targeting abnormality on mammogram**	13 (14.44%)	3/13 (23.07%)
**Diffuse abnormality on US**	10 (11.11%)	7/10 (70%)
**Retroareolar biopsies to assess feasibility for skin sparing mastectomy**	2 (0.02%) (2.22%)	0/2 (0%)
**Total**	90	28/90 (31.11%)

### Non-mass enhancement detected on MRI

**Figure 2:**
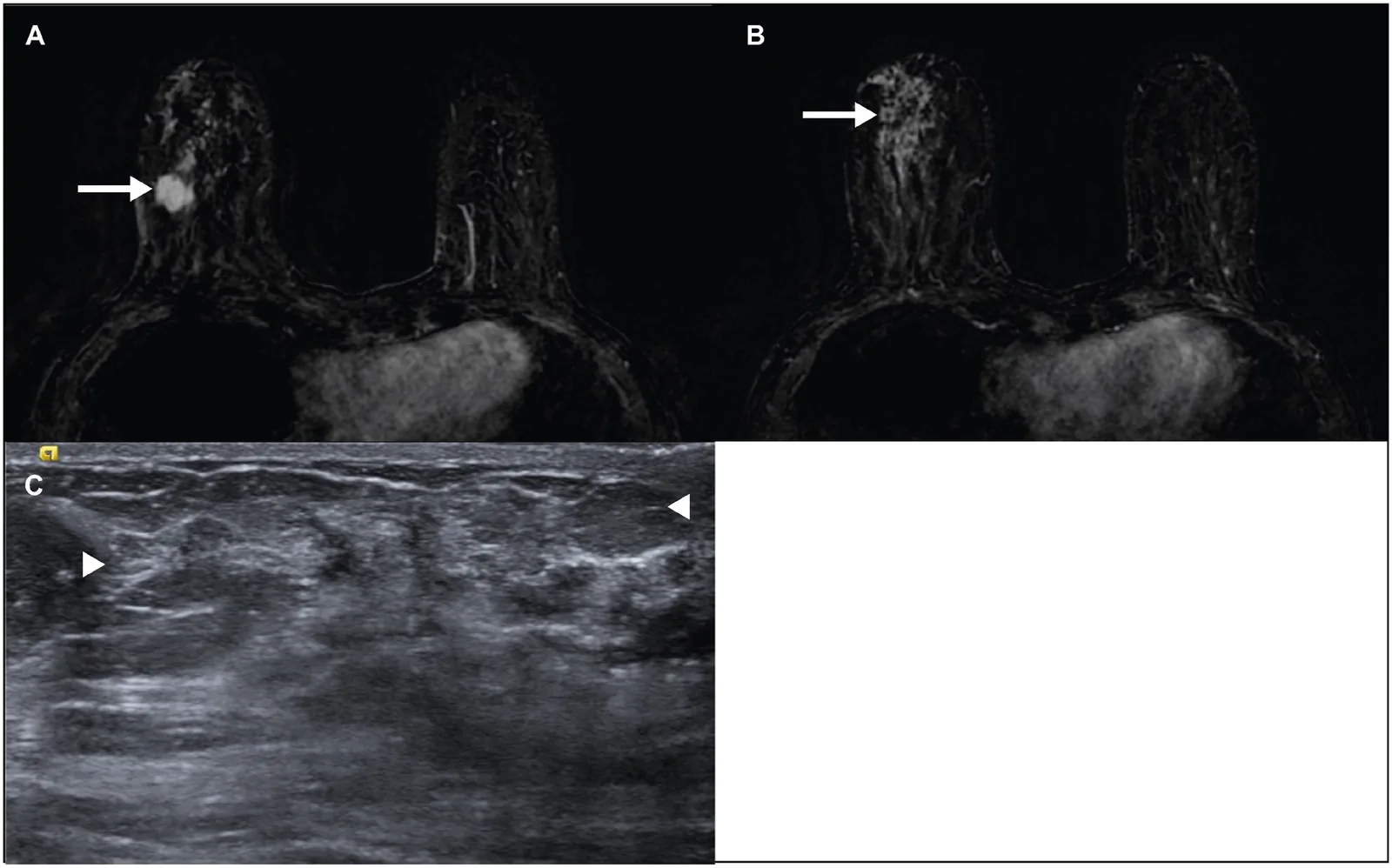
(A) 49-year-old with right breast Grade 3 invasive carcinoma mixed NST and lobular features (arrow). (B) Staging MRI demonstrated extensive NME with a clustered ring appearance anterior and slightly superior to the known tumor mass highly suspicious for DCIS. Mammogram of this area was normal. (C) At second look US right breast anterior and superior to the known malignancy there was mild hypoechoic and fibrocystic change but no discrete lesion identified. This area was felt to correspond to the area of NME seen on MRI and a fan of biopsy of the area was obtained. Histology confirmed high grade Ductal carcinoma in situ (B5a).

The most common indication for fan biopsy was to sample MRI detected non-mass enhancement (NME) (44/90; 49% of cases), with MRI originally performed for local staging of breast cancer (34/44) or as a problem-solving tool (10/44) (Figure 2). Histology in 11/44 cases yielded a B5 result; 1 was B4; 6 were B3 and 26 were benign. All cases where the original MRI abnormality had been classified as suspicious (MRI score at least 4)^7^ with a B1-B2 result went on to have MRI-guided biopsy (7/26) and in 3 patients this resulted in an upgrade (1 case was upgraded from B1 to B5a and 2 from B2 to B3). Final outcomes are recorded in Flowchart 1.

**Figure:**
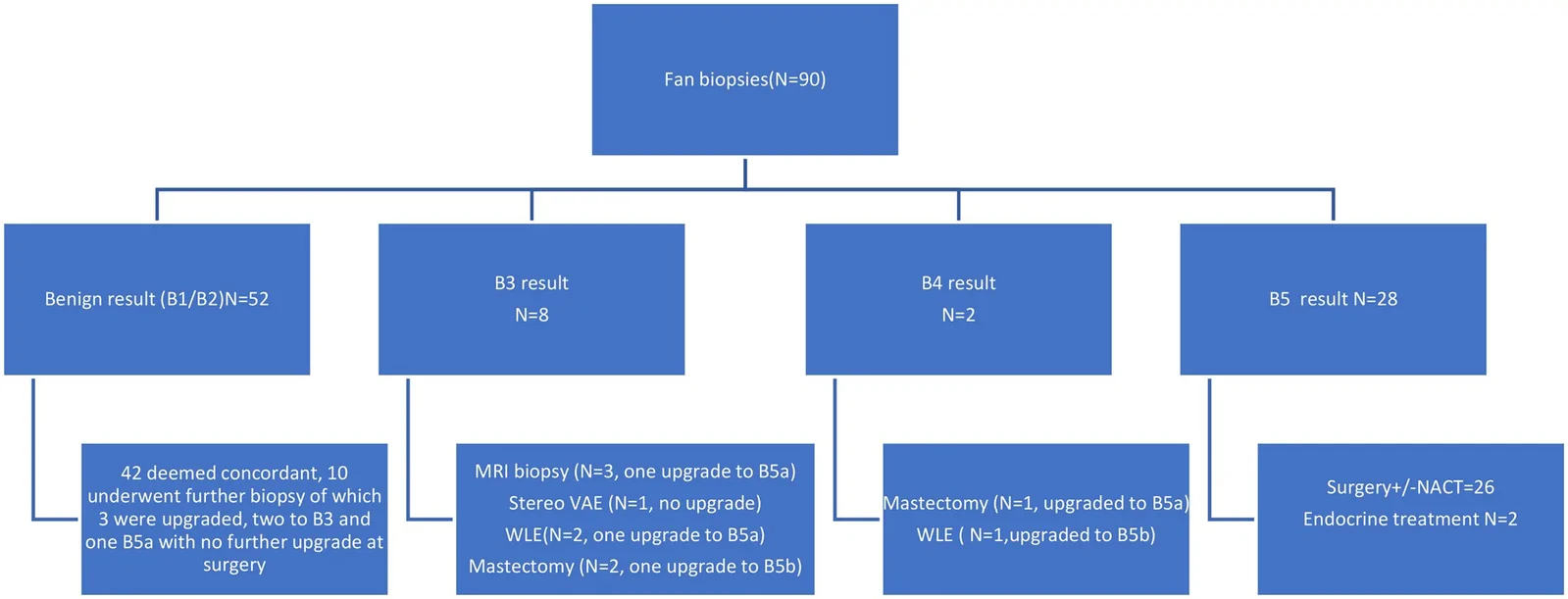
**Flowchart 1.** Biopsy results and subsequent management.

### Clinical abnormality with vague or no US changes

**Figure 3:**
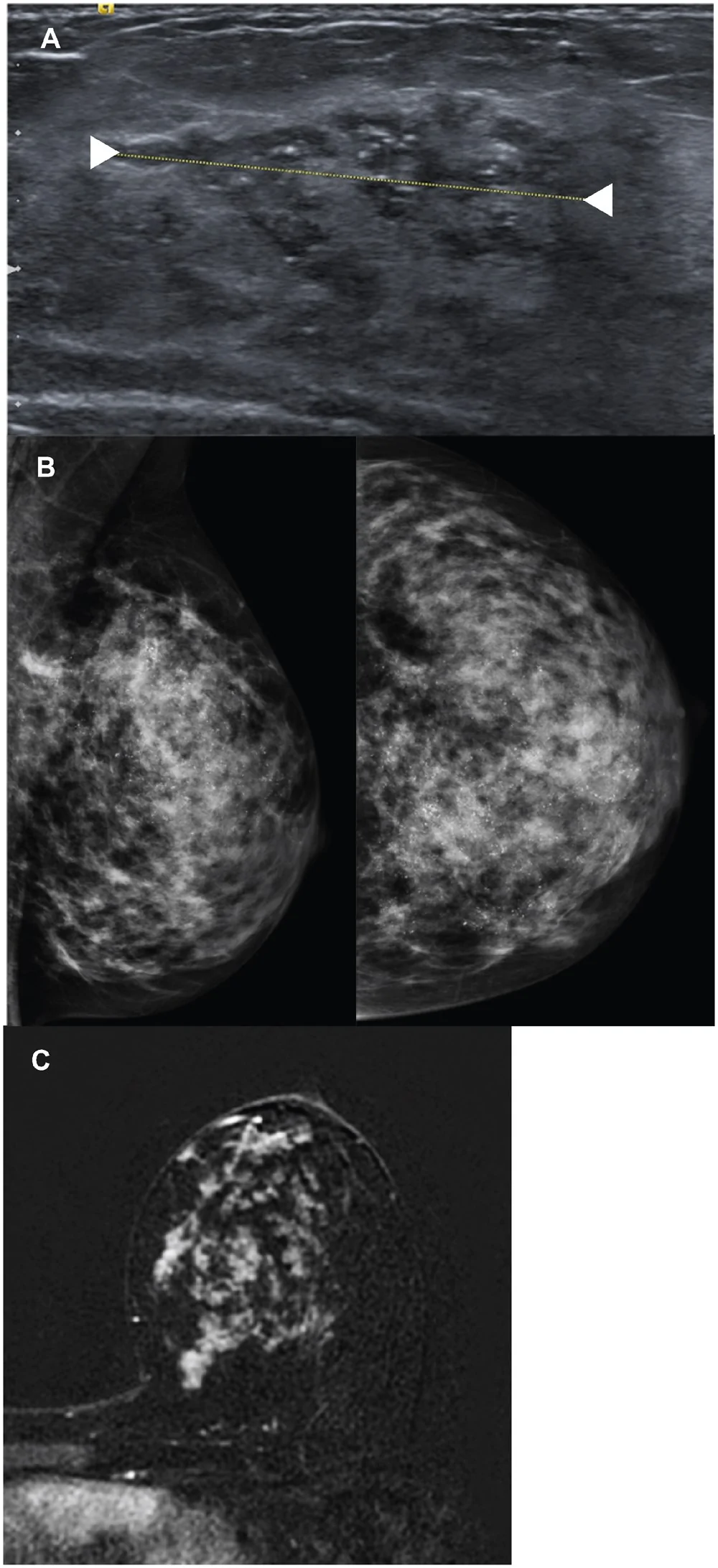
A 33-year-old with clinically palpable E2 nodularity left upper breast. (A) US image of the area of clinical concern shows area of altered echogenicity and scattered microcalcifications. Fan biopsy was performed through the area. Histology showed high grade DCIS with focal microinvasion (B5a). (B) Subsequent mammogram showed extensive microcalcification and (C) MRI showed abnormal NME throughout the left breast.

The second most common indication for performing fan biopsy (21/90; 23% of cases) was to sample a predominantly clinical abnormality (Figure 3). 7/21 biopsies resulted in a diagnosis of malignancy, 13/21 were benign and one patient had a B3 result (upgraded to B5a at surgery).

### Abnormality on mammogram

**Figure 4:**
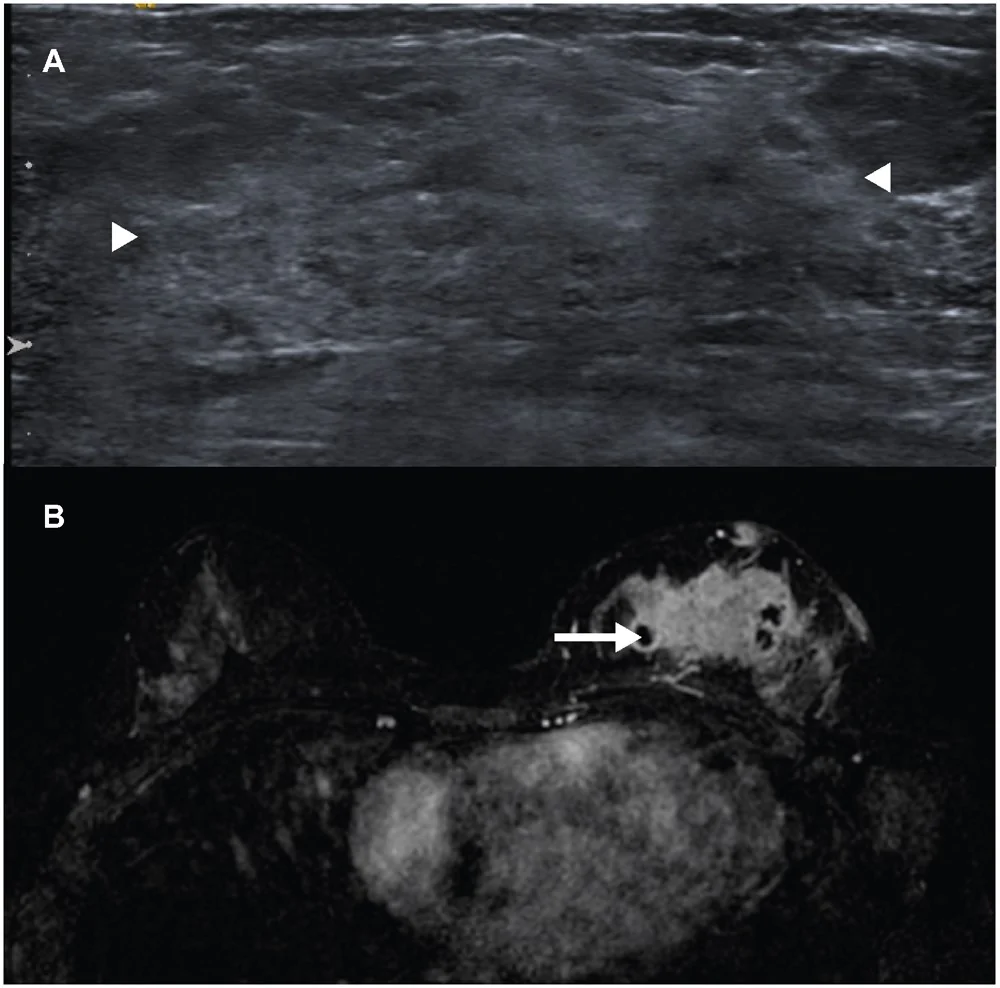
46-year-old with large clinically palpable lump left breast. (A) US image shows diffuse nonspecific echogenic change throughout the left upper breast. Fan biopsy was performed. Initial histopathology: Florid nonspecific active chronic mastitis with focal granulomatous component (B2). (B) Subsequent MRI shows corresponding abnormal enhancement throughout the left upper breast with cystic spaces. Further lab investigations confirmed idiopathic granulomatous mastitis.

A third indication was to sample mammographic abnormalities without a discrete sonographic mass (13/90; 14%) (Figure 4). Most cases (9/13) targeted microcalcifications with subtle hypoechoic change or were performed as an alternative to x-ray guidance due to technical limitations (eg, retroareolar or very posterior location), patient intolerance, or pregnancy. Histology demonstrated malignancy in 3 cases (2 B5a, 1 B5b) and benign findings in 6. Two biopsies were non-diagnostic due to absence of calcification on specimen radiography and subsequently underwent stereotactic vacuum-assisted biopsy or surgical excision, confirming benign pathology. The remaining cases (4/13) targeted subtle sonographic correlates for mammographic asymmetry (Figure 4) or distortion, yielding 2 benign results, 1 B3 (no upgrade at VAE), and 1 B4 lesion, result (upgraded to B5a at surgery).

### Diffuse abnormality on US

**Figure 5:**
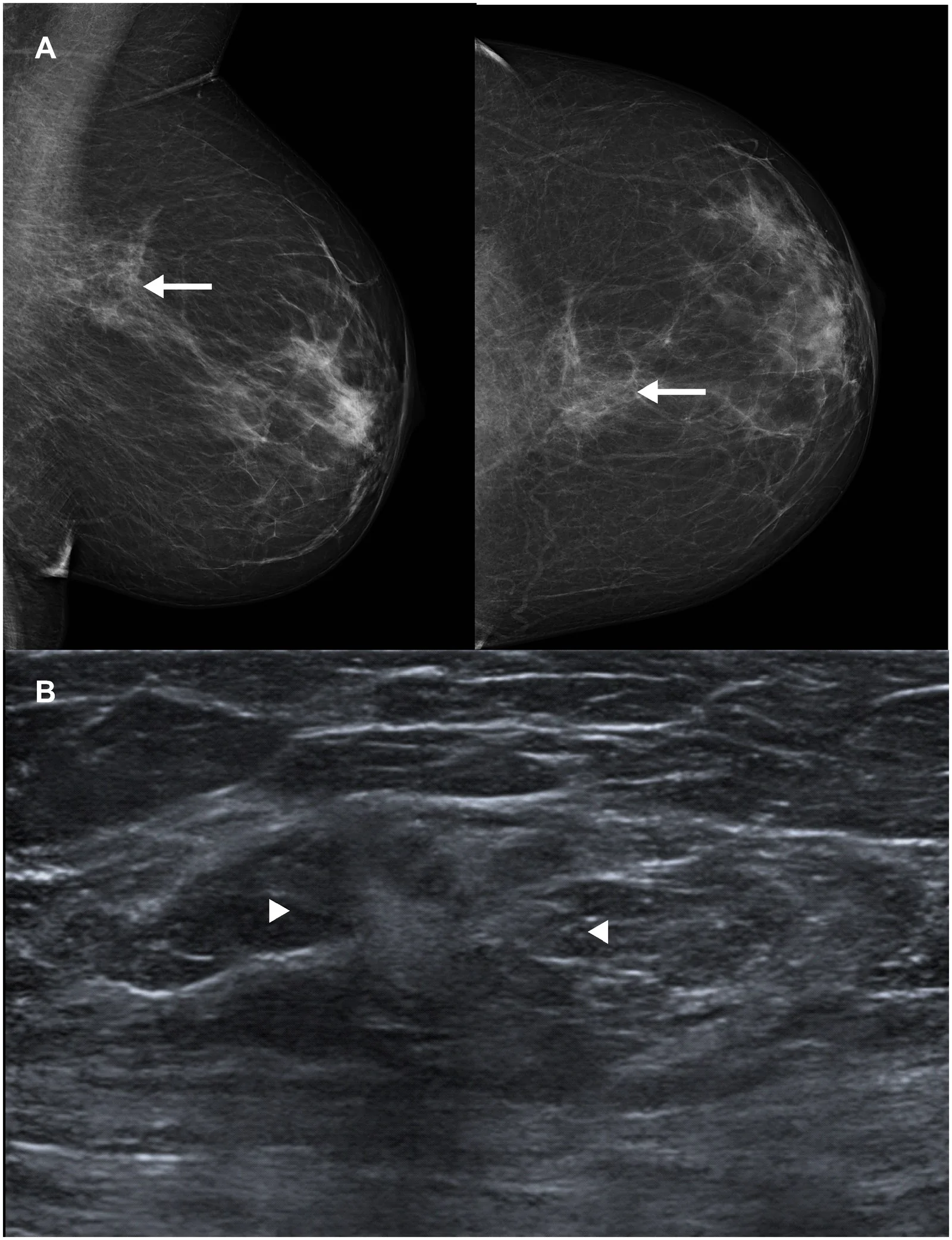
73-year-old female present with E3 lump in the left breast. (A) Left mammogram shows an asymmetric density in the posterior central breast (arrows) with no prior MG at the time. (B) US image shows glandular tissue with vague echogenic change. Fan biopsy of the area was performed in view of clinical concern. Histopathology: Invasive lobular carcinoma, Grade 2, ER+PR +HER2-.

The fourth indication (10/90; 11% cases) was to sample a diffuse abnormality on US (Figure 5). The majority of these biopsies (7/10) yielded a malignant result; 2/10 proved mastitis and in one fan biopsy performed in a male patient histology confirmed gynecomastia with PASH.

### Surgical planning

2/90 cases were retro-areolar fan biopsies performed at the surgeon’s request to assess feasibility for nipple- sparing mastectomy which yielded B1 results, both confirmed at surgery.

Fan biopsy histology and patient management: Table 3 lists the histology results and Flowchart 1 illustrates the biopsy results and subsequent management.

**Table 3:** Histology results from US-guided fan biopsy breast.

	Number of cases
**B1**	21 (23.33%)
**B2**	31 (34.44%)
**Fibrocystic change**	17
**Inflammation**	6
**Fat necrosis**	3
**Others** ^a^	5
**B3**	8 (8.88%)
**AIDEP**	4
**Lobular neoplasia**	3
**Radial scar without atypia**	1
**B4**	2 (2.22%)
**B5**	28 (31.11%)
**Invasive cancer NST**	11
**Invasive lobular cancer**	4
**Invasive micropapillary cancer**	1
**High grade DCIS**	9
**Intermediate/low grade DCIS**	2
**Pleomorphic LCIS**	1

a Includes scar tissue (*N* = 2), gynecomastia (*N* = 1), lactational change (*N* = 1), and sclerosed fibroadenoma (*N* = 1).

A malignant result (B5) was obtained in 28/90 cases (31%). The majority of these biopsies (11/28) were performed to target an abnormality identified on MRI, as described above. Eight biopsies were performed to target a diffuse abnormality on US, 7/28 were clinically directed (Figure 5) and 2 were performed to target microcalcification.

A B4 result was obtained in 2 cases. One of these patients opted for a mastectomy due to the presence of ipsilateral biopsy proven DCIS in a different quadrant with upgrade to B5a and the second patient opted for a diagnostic excision at the time of mastectomy for contralateral breast cancer with upgrade to B5b.

**Figure 6:**
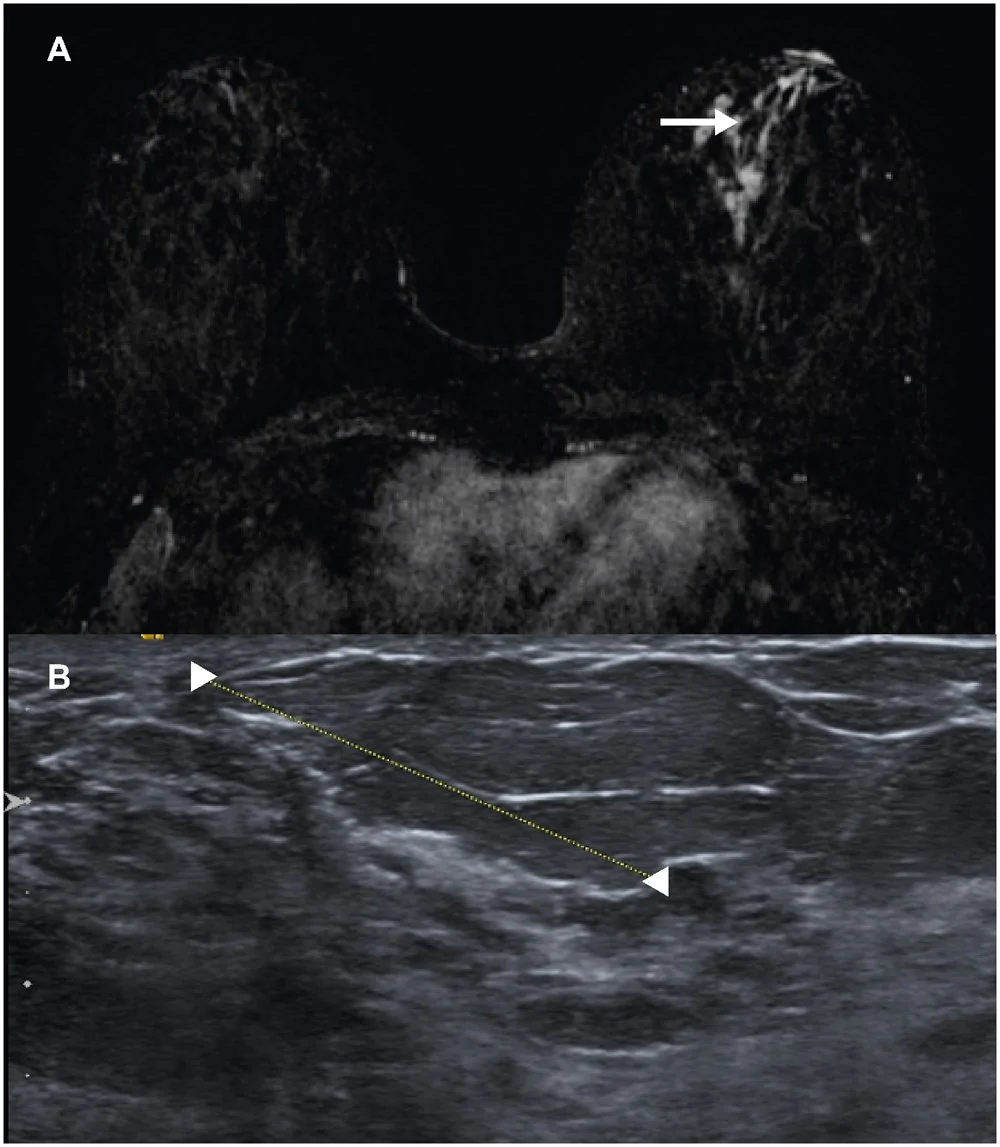
56-year-old with Paget’s disease of the left nipple on skin biopsy with normal mammogram. (A) MRI image shows NME extending from the left nipple towards the center of the breast (arrow) highly suspicious for DCIS. (B) Second look US shows corresponding subtle hypoechoic change posterior to the left nipple. A fan biopsy was performed. Histopathology: Duct ectasia, fibrocystic change, columnar cell changes and LCIS. (B3) Subsequently upgraded to high grade DCIS on left central excision.

A B3 result was observed in 8 cases, all of which underwent further procedures. Three lesions underwent MRI-guided vacuum biopsy, one underwent stereotactic-guided VAE, and 2 underwent surgical diagnostic excision (including one central duct excision for concurrent Paget’s disease [Figure 6]). Two patients opted for mastectomy—one due to ipsilateral malignancy and the other, a high-risk patient with contralateral malignancy, chose prophylactic mastectomy on the side of the B3 lesion. Three cases were eventually upgraded: 1 to B5b and 2 to B5a (Flowchart 1).

Histology results were benign (B1/B2) in 52 patients. In 42 cases, the results were accepted as concordant following MDT discussion, and went on to have repeat clinical examination, follow-up breast MRI at 6 months as deemed necessary, or treatment for concurrent breast malignancy.

10 patients with discordant results, underwent a further biopsy as per MDT decision (4 underwent MRI-guided biopsy, 1 underwent stereotactic VAB and 5 patients underwent surgical diagnostic excision). Of these, patients 3/10 (30%) who underwent MRI-guided biopsy were subsequently upgraded (2 to B3 and 1 to B5a) with no further upgrade in the B3 cases.

Diagnostic accuracy was assessed by correlating fan biopsy histology with subsequent histology or imaging follow-up (Table 4). Ten cases lost to follow-up were excluded from analyses of diagnostic performance. The sensitivity, specificity, positive predictive value (PPV), negative predictive value (NPV), and overall accuracy are summarized in Table 5.

**Table 4:** Correlation of results of US-guided fan biopsy histology with subsequent histology and follow up findings.^a^

Fan biopsy result	Subsequent biopsy or follow up findings		Total
	Malignant	Benign	
**Positive**	33	7	40
**Negative**	4	36	40
**Total**	37	43	80

a Ten cases lost to follow-up were excluded from this analysis.

**Table 5:** Diagnostic performance of US-guided fan biopsy in the eligible subset.

Metric	Estimate	95% CI
**Sensitivity**	89.2%	74.6%-97.0%
**Specificity**	83.7%	69.3%-93.2%
**PPV**	82.5%	67.2%-92.7%
**NPV**	90.0%	76.3%-97.2%
**Accuracy**	86.2%	76.7%-92.9%

## Discussion

This study describes the commonly used but infrequently documented technique of US-guided non-focused breast “fan” biopsy. The most common indications for use of this technique in our experience were to target NME originally found on MRI, followed by clinical, mammographic or diffuse US abnormality. Our results show that US-guided fan biopsy was useful to guide the next step in the patient’s pathway in 93% (84/90) cases with 4 false negative and 2 inconclusive results. To our knowledge, this is the first report on the utility of US-guided fan biopsy of the breast despite anecdotal evidence of its use in breast units across the UK.

The majority of fan biopsies in our study were performed to sample MRI-identified abnormalities. Several studies have examined second-look US to identify MRI lesions, and it is widely accepted that lesions most likely to have a US correlate are masses rather than NME, larger in size, and with a higher level of suspicion.^8–14^ Careful review of the MRI prior to second-look US is important, considering differences in patient positioning during prone MRI.^4^^,^^8^ Trop et al. used the nipple, landmarks, and zones to locate lesions,^15^ while Peter et al. used the nipple as a reference, counting sections above/below the nipple for vertical position and medial/lateral distance for greater accuracy and shorter scanning times.^16^

**Figure 7:**
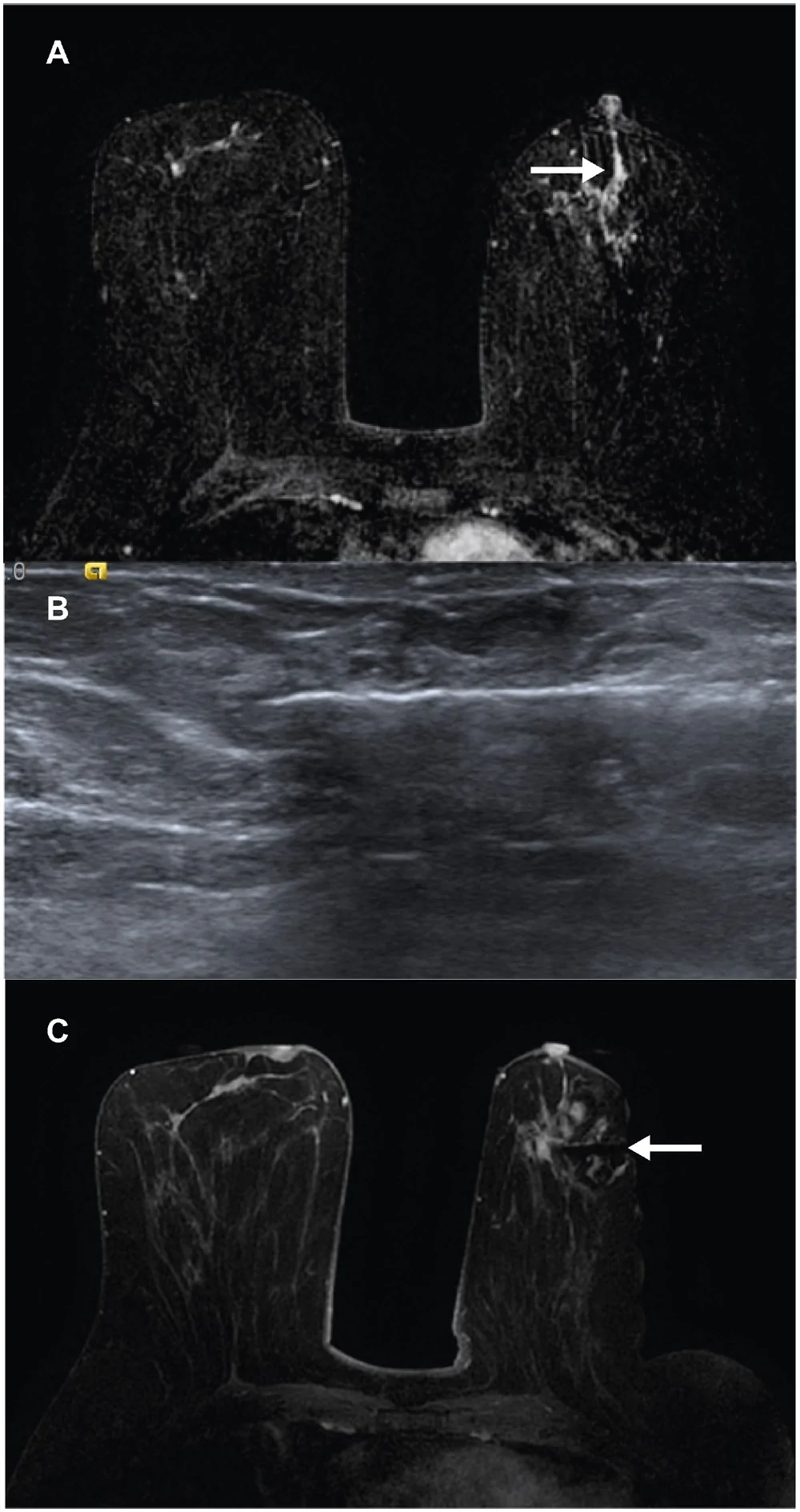
65-year-old with known cancer left upper outer breast. (A) Staging MRI showed extensive NME in the left breast including retroareolar area. Fan biopsy recommended to establish extent of disease. (B) US was normal, US- guided retro areolar fan biopsy was performed in view of MRI findings, (tip of needle visualized in situ) which yielded a B1 result which was discordant. (C) MRI guided vacuum assisted biopsy targeting retroareolar enhancement. Arrow shows MRI biopsy introducer needle in good position. Histopathology: High grade DCIS (B5a).

One potential concern with fan biopsy is “random” sampling that may miss low-volume disease. In our experience, 4 fan biopsies targeting MRI abnormalities were false negatives, with subsequent biopsy or surgery revealing histological upgrades. One biopsy targeting NME contiguous with known cancer yielded a B1 result, but follow-up MRI-guided biopsy confirmed malignancy (B5a) (Figure 7). In 2 cases, fan biopsy showed B2 results, while subsequent MRI-guided biopsy upgraded pathology to B3 without further upgrade later. In each of these cases, despite reassuring fan biopsy results, the original MRI-derived suspicion prompted additional sampling. In a fourth case, fan biopsy targeting NME in a patient with metastatic axillary nodes yielded a B1 result; subsequent mastectomy revealed cancer, though its relation to the biopsy site was unclear. Thus, in only 2 of these cases did the fan biopsy miss malignancy.

**Figure 8:**
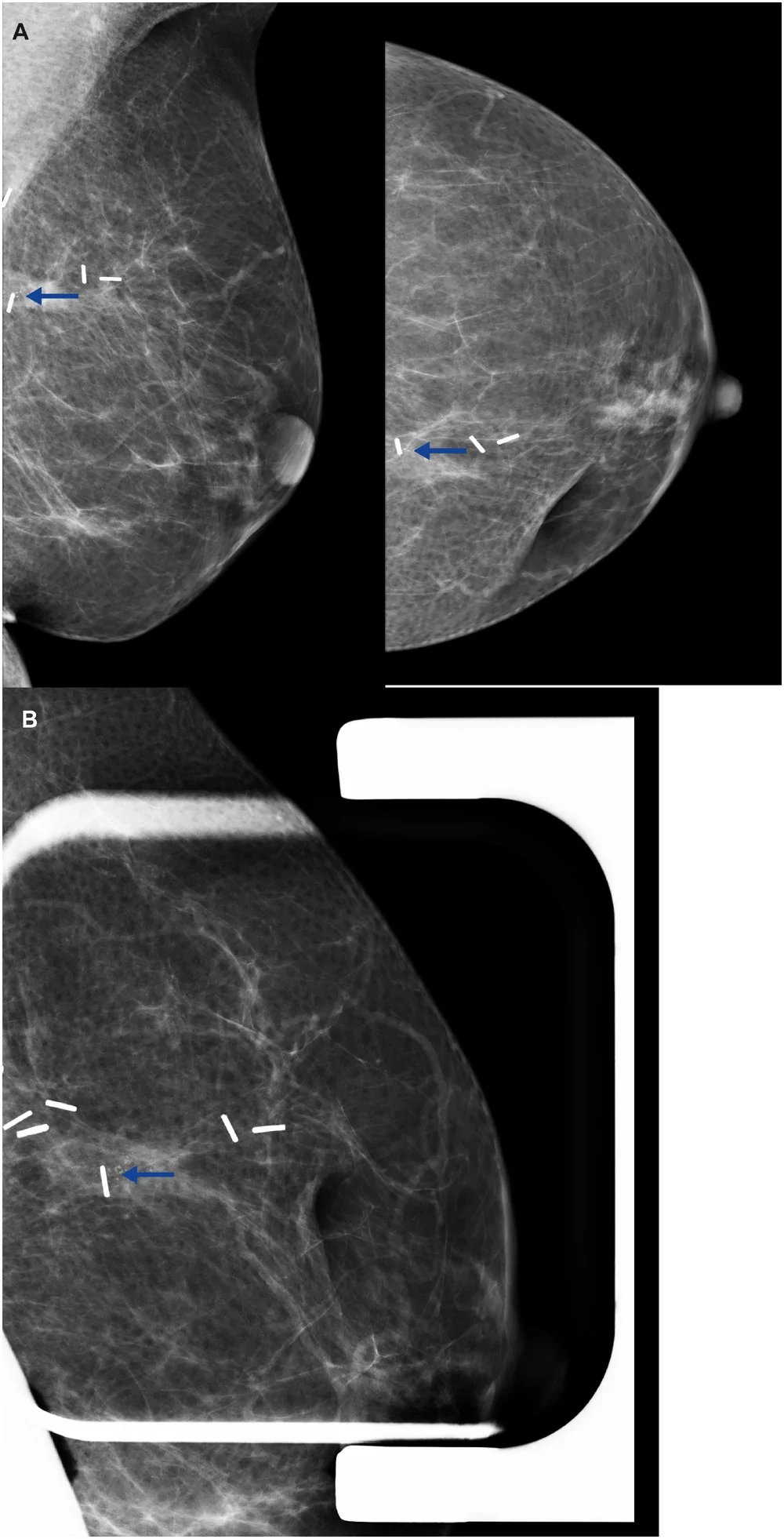
60-year-old with new indeterminate microcalcification adjacent to surgical clips from previous WLE on screening mammogram. (A) Left mammogram and (B) magnification view of the left breast show indeterminate microcalcification (blue arrow) around surgical clips from previous WLE. Attempts at stereotactic vacuum assisted biopsy were unsuccessful due to very posterior location of the microcalcification. Therefore, an ultrasound was performed, surgical clips were identified and a fan of 6 × 14G biopsies were undertaken around the clips. Histology was benign (B2) although no calcification was seen on specimen x-ray. Subsequent wire-guided WLE confirmed scar, fat necrosis, and stromal microcalcification; no evidence of malignancy B2.

**Figure 9:**
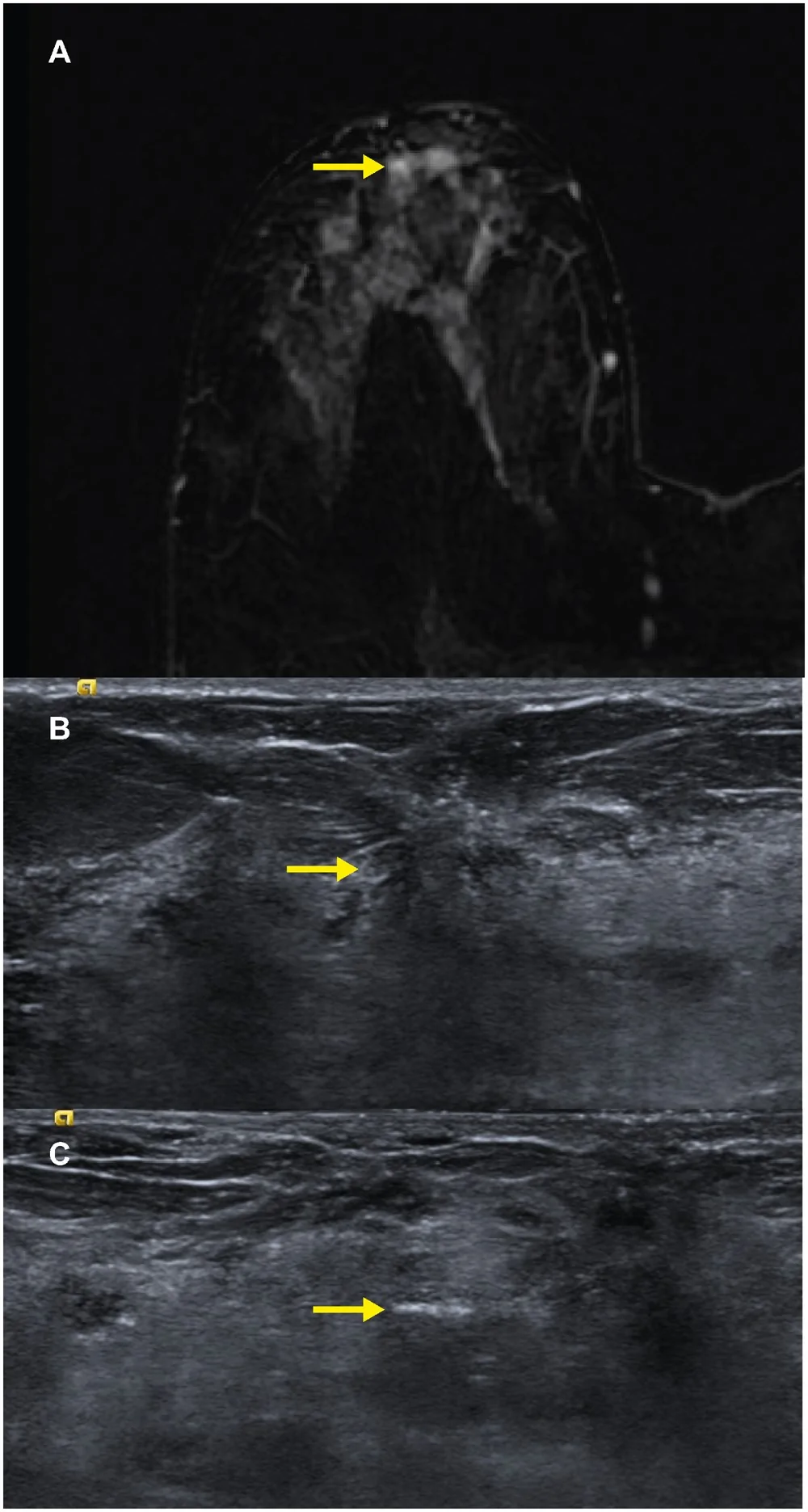
59-year-old with invasive lobular carcinoma left breast. (A) Staging MRI showed a contralateral focal area of enhancement (arrow) just superior to the right nipple—MRI 4. MRI guided biopsy was not technically feasible due to proximity of the lesion to the skin and nipple. (B) Second look US right breast showed a subtle area of echogenic change and distortion. (C) A fan of biopsies was taken through this area and a marker clip deployed (arrow). Histology: Atypical lobular/intraductal epithelial proliferation with mixed features of LCIS and low-grade DCIS, best regarded as B4. Histology from diagnostic excision performed at the same time as WLE surgery for contralateral malignancy confirmed multifocal mixed invasive NST and lobular carcinoma, Grade 2, ER+PR+Her2-.

X-ray-guided VAB, with stereotactic or tomosynthesis guidance, is well accepted as the standard technique to biopsy a mammographic abnormality.^17–20^ During our study period, US-guided fan biopsy was performed in a limited number of cases to target a mammographic abnormality and the majority of these fan biopsies were helpful, however, 2 were deemed non-diagnostic as no calcium was seen on the specimen x-ray did and these patients went on to have further sampling which confirmed benign histology results. Fan biopsy was found particularly helpful in challenging cases where stereotactic and MRI-guided biopsies were not technically feasible (Figures 8 and 9).

The diagnostic value of clinically guided core biopsy in the diagnosis of breast malignancy in palpable clinically suspicious lesions is well established.^21^^,^^22^ In our study, US-guided fan biopsy helped establish malignancy in 33% of patients (7/21 patients), performed for a predominantly palpable clinical abnormality.

A high proportion of fan biopsies during the study period in cases with diffuse US abnormality (70%) yielded a malignant result. In cases such as inflammatory breast cancer, diagnosis can be challenging because of diffusely increased density, stromal coarsening, and skin thickening.^23^^,^^24^ Wider sampling through the use of US-guided fan biopsy can help avoid false negative results.

If histology from fan biopsy confirms malignancy, this enables discussion of treatment options including surgical planning, with minimal delay. If the histology is benign, careful review of prior imaging is required to ensure clinical, radiological and pathological concordance and due importance should be given to the original level of suspicion. Clip placement and imaging should be performed to confirm that the marker clip lies within the abnormality on the original imaging, that is, mammogram or MRI and if this does not correlate correctly, re-biopsy or follow-up is warranted.^1–4^^,^^8^

In our unit, we find fan biopsy to be well tolerated by patients with no difference in acceptability between this technique and conventional targeted core biopsy. It is quick to perform and no complications were reported.

Although a detailed cost analysis is outside the scope of this study it is likely that this technique would prove cost effective particularly in instances when x-ray and MRI-guided biopsy can be avoided. Alternative techniques have been explored to improve lesion localization when no sonographic correlate is identified. Real-time MRI–US fusion imaging has shown improved detection at second-look ultrasound but requires dedicated software and, in many cases, additional supine MRI acquisition, limiting its practicality.^25–28^ Automated breast ultrasound (ABUS) has also demonstrated promising results in this setting but remains limited by availability.^29^^,^^30^ Contrast-enhanced mammography-guided biopsy is an emerging alternative that integrates with existing mammographic systems and may offer improved patient tolerance compared to MRI, although further evidence is needed to establish its role and efficacy.^31–33^

Fan biopsy relies on accurate anatomical correlation, considering differences in positioning between US and mammography or MRI, and using anatomical landmarks where possible for a systematic regional sampling. Thus, it adds value by providing an alternative means of obtaining histology without additional sophisticated equipment.

The main limitations of this retrospective study are the small sample size and absence of long-term follow-up following benign concordant biopsies. All cases meeting clinical criteria were included, but because biopsy was performed based on clinical need, selection bias cannot be excluded. Ten cases (11%) were lost to follow-up and excluded from the diagnostic performance analysis, this may introduce attrition bias. To the best of our knowledge, there is no literature on this technique, however, using the search term “fan biopsy” may not have captured all articles relating to this to topic. US images are subjective and often more apparent in real-time, particularly true for areas of diffuse textural change where targeting relies on dynamic imaging. Thus, images were not re-reviewed for the study and the original opinion of the imager was recorded. Although interobserver variability was not studied, all biopsies were performed by experienced operators and results reviewed at MDT.

In conclusion, achieving an accurate histological diagnosis is possible through US-guided fan biopsy which can avoid delays and facilitate treatment decisions. Our results suggest that this relatively simple procedure has potential to speed up diagnosis and perhaps reduce costs in instances where it prevents referral for MRI-guided biopsy, and therefore should be considered in selected cases as a means to obtain histology. Further large-scale prospective research is needed to establish the full efficacy of this technique.

## References

[1] American College of Radiology. ACR Practice Parameter for the Performance of Ultrasound-Guided Percutaneous Breast Interventional Procedures. Revised 2021. Accessed November 2024. https://www.acr.org/-/media/ACR/Files/Practice-Parameters/US-Breast.pdf

[2] Mahoney MC , Newell MS. Breast intervention: how I do it. Radiology. 2013;268:12-24. 10.1148/radiol.13120985 23793589

[3] Chesebro AL , Chikarmane SA, Ritner JA, Birdwell RL, Giess CS. Troubleshooting to overcome technical challenges in image-guided breast biopsy. Radiographics. 2017;37:705-718. 10.1148/rg.2017160117 28410063

[4] Papalouka V , Kilburn-Toppin F, Gaskarth M, Gilbert F. MRI-guided breast biopsy: a review of technique, indications and radiological-pathological correlations. Clin Radiol. 2018;73:908.e17–908.e25. 10.1016/j.crad.2018.05.029 30041954

[5] Lee A , Pinder S. An overview of B coding of breast core biopsy categorisation and management implications. Diagn Histopathol. 2024;30:132-140. 10.1016/j.mpdhp.2023.11.004

[6] Brenner RJ , Bassett LW, Fajardo LL, et al Stereotactic core-needle breast biopsy: a multi-institutional prospective trial. Radiology. 2001;218:866-872. 10.1148/radiology.218.3.r01mr44866 11230668

[7] Maxwell AJ , Ridley NT, Rubin G, et al The Royal College of Radiologists Breast Group breast imaging classification. Clin Radiol. 2009;64:624-627. 10.1016/j.crad.2009.01.010 19414086

[8] Park VY , Kim MJ, Kim EK, Moon HJ. Second-look US: how to find breast lesions with a suspicious MR imaging appearance. Radiographics. 2013;33:1361-1375. 10.1148/rg.335125109 24025929

[9] Meissnitzer M , Dershaw DD, Lee CH, Morris EA. Targeted ultrasound of the breast in women with abnormal MRI findings for whom biopsy has been recommended. AJR Am J Roentgenol. 2009;193:1025-1029. 10.2214/AJR.09.2480 19770325

[10] Chikarmane SA , Jin B, Giess CS. Accuracy of MRI-directed ultrasound and subsequent ultrasound-guided biopsy for suspicious breast MRI findings. Clin Radiol. 2020;75:185-193. 10.1016/j.crad.2019.10.013 31767141

[11] Bick U , Trimboli RM, Athanasiou A, et al Image-guided breast biopsy and localisation: recommendations for information to women and referring physicians by the European Society of Breast Imaging. Insights Imaging. 2020;11:12. 10.1186/s13244-019-0803-x 32025985 PMC7002629

[12] Carbognin G , Girardi V, Calciolari C, et al Utility of second-look ultrasound in the management of incidental enhancing lesions detected by breast MR imaging. Radiol Med. 2010;115:1234-1245. 10.1007/s11547-010-0561-9 20574702

[13] Spick C , Baltzer PA. Diagnostic utility of second-look US for breast lesions identified at MR imaging: systematic review and meta-analysis. Radiology. 2014;273:401-409. 10.1148/radiol.14140474 25119022

[14] Bumberger A , Clauser P, Kolta M, et al Can we predict lesion detection rates in second-look ultrasound of MRI-detected breast lesions? a systematic analysis. Eur J Radiol. 2019;113:96-100. 10.1016/j.ejrad.2019.02.008 30927966

[15] Trop I , Labelle M, David J, Mayrand MH, Lalonde L. Second-look targeted studies after breast magnetic resonance imaging: practical tips to improve lesion identification. Curr Probl Diagn Radiol. 2010;39:200-211. 10.1067/j.cpradiol.2009.07.006 20674767

[16] Peter P , Dhillon R, Bose S, Bourke A. MRI screening-detected breast lesions in high-risk young women: the value of targeted second-look ultrasound and imaging-guided biopsy. Clin Radiol. 2016;71:1037-1043. 10.1016/j.crad.2016.03.009 27083056

[17] Huang ML , Adrada BE, Candelaria R, Thames D, Dawson D, Yang WT. Stereotactic breast biopsy: pitfalls and pearls. Tech Vasc Interv Radiol. 2014;17:32-39. 10.1053/j.tvir.2013.12.006 24636329

[18] Wilkinson L , Thomas V, Sharma N. Microcalcification on mammography: approaches to interpretation and biopsy. Br J Radiol. 2017;90:20160594. 10.1259/bjr.20160594 27648482 PMC5605030

[19] Bae S , Yoon JH, Moon HJ, Kim MJ, Kim EK. Breast microcalcifications: diagnostic outcomes according to image-guided biopsy method. Korean J Radiol. 2015;16:996-1005. 10.3348/kjr.2015.16.5.996 26357494 PMC4559796

[20] Kim TE , Kim DB, Jung JH, Lee EK. Sonographic visibility and feasibility of biopsy under ultrasound guidance of suspicious microcalcification-only breast lesions: a single-centre study. Hong Kong J Radiol. 2015;18:125-133. 10.12809/hkjr1514264

[21] Alameer A , Common M, Elwahab SA, et al Clinically guided core biopsy and cutaneous punch biopsy in the evaluation of breast lesions: a necessary test or an obsolete skill? Ir J Med Sci. 2023;192:317-319. 10.1007/s11845-022-02937-8 35132568 PMC9892099

[22] Hari S , Kumari S, Srivastava A, Thulkar S, Mathur S, Veedu PT. Image-guided versus palpation-guided core needle biopsy of palpable breast masses: a prospective study. Indian J Med Res. 2016;143:597-604. 10.4103/0971-5916.187108 27488003 PMC4989833

[23] An YY , Kim SH, Cha ES, et al Diffuse infiltrative lesion of the breast: clinical and radiologic features. Korean J Radiol. 2011;12:113-121. 10.3348/kjr.2011.12.1.113 21228947 PMC3017875

[24] Yeh ED , Jacene HA, Bellon JR, et al What radiologists need to know about diagnosis and treatment of inflammatory breast cancer: a multidisciplinary approach. Radiographics. 2013;33:2003-2017. 10.1148/rg.337135503 24224593

[25] Park AY , Seo BK, Han H, et al Clinical value of real-time ultrasonography–MRI fusion imaging for second-look examination in preoperative breast cancer patients: additional lesion detection and treatment planning. Clin Breast Cancer. 2018;18:261-269. 10.1016/j.clbc.2017.07.007 28774783

[26] Mazzei MA , Di Giacomo L, Fausto A, Gentili F, Mazzei FG, Volterrani L. Efficacy of second-look ultrasound with MR coregistration for evaluating additional enhancing lesions of the breast: review of the literature. Biomed Res Int. 2018;2018:3896946. 10.1155/2018/3896946 30420960 PMC6215588

[27] Sakakibara J , Nagashima T, Fujimoto H, Takada M, Ohtsuka M. A review of MRI (CT)/US fusion imaging in treatment of breast cancer. J Med Ultrason (2001). 2023;50:367-373. 10.1007/s10396-023-01316-9 37231224 PMC10354153

[28] Nakashima K , Uematsu T, Harada TL, et al MRI-detected breast lesions: clinical implications and evaluation based on MRI/ultrasonography fusion technology. Jpn J Radiol. 2019;37:685-693. 10.1007/s11604-019-00866-8 31486968

[29] Chae EY , Shin HJ, Kim HJ, et al Diagnostic performance of automated breast ultrasound as a replacement for a hand-held second-look ultrasound for breast lesions detected initially on magnetic resonance imaging. Ultrasound Med Biol. 2013;39:2246-2254. 10.1016/j.ultrasmedbio.2013.07.005 24035627

[30] Kim Y , Kang BJ, Kim SH, Lee EJ. Prospective study comparing two second-look ultrasound techniques: handheld ultrasound and an automated breast volume scanner. J Ultrasound Med. 2016;35:2103-2112. 10.7863/ultra.15.11076 27503758

[31] Sammarra M , Piccolo CL, Sarli M, et al Contrast-enhanced mammography-guided biopsy: preliminary results of a single-centre retrospective experience. J Clin Med. 2024;13:933. 10.3390/jcm13040933 38398247 PMC10889410

[32] Alcantara R , Posso M, Pitarch M, et al Contrast-enhanced mammography-guided biopsy: technical feasibility and first outcomes. Eur Radiol. 2023;33:417-428. 10.1007/s00330-022-09021-w 35895121 PMC9755098

[33] Alcantara R , Azcona J, Pitarch M, Vall E, Vila-Trias E, Arenas EN. Contrast-enhanced mammography-guided biopsy: principles, challenges and opportunities. Insights Imaging. 2025;16:262. 10.1186/s13244-025-02148-6 41284217 PMC12644378

